# Supplementary material for: Translation of non-standard codon nucleotides reveals minimal requirements for codon-anticodon interactions
Source: Nat Commun. 2018 Nov 19;9:4865. doi: 10.1038/s41467-018-07321-8 (PMC6242847; doi:10.1038/s41467-018-07321-8)
Supplement: Supplementary file 1 — Supplementary Information [file 41467_2018_7321_MOESM1_ESM.pdf]

Supplementary Information

Translation of non-standard nucleotides reveals minimal requirements for codon-anticodon interactions

Hoernes et al.

Supplementary Figure 1

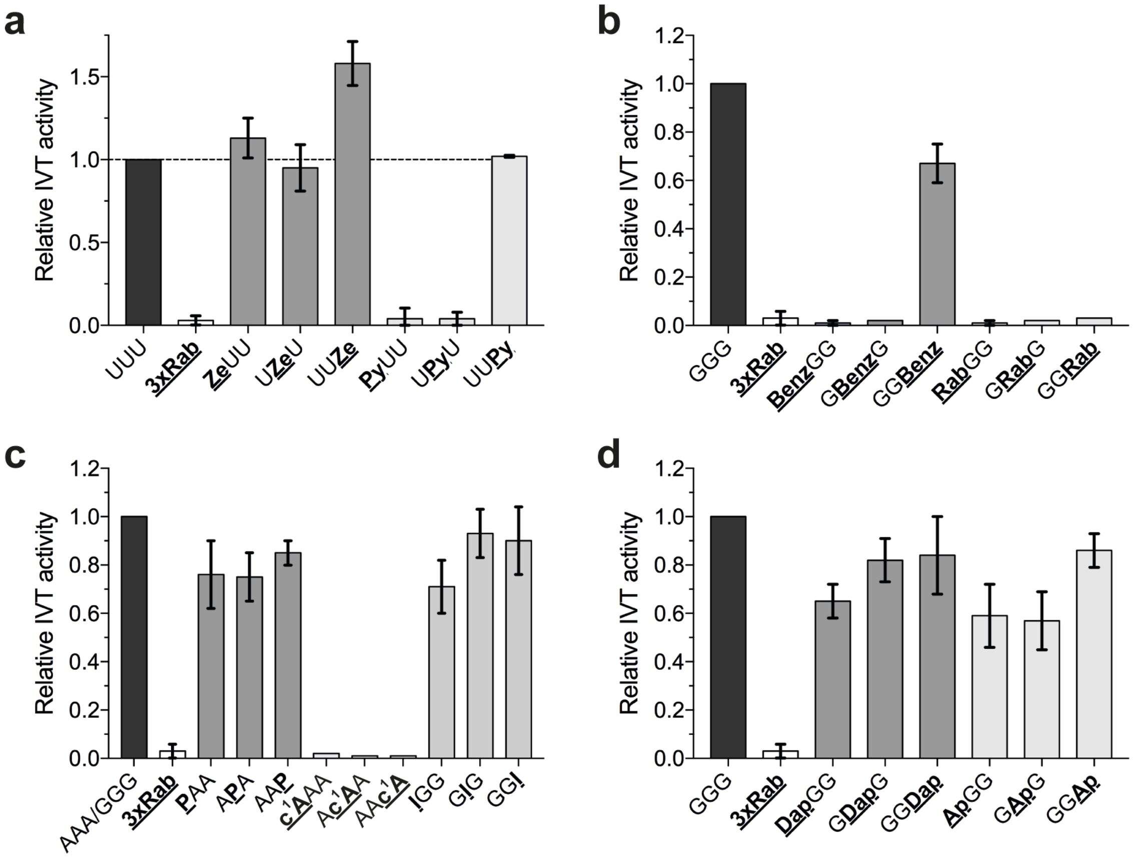

Supplementary Figure 1: Translation of modified sense codons in the PURExpress *in vitro* translation system. The efficiency of translation of **(a)** zebularine (Ze) and pyridone (Py) **(b)** benzimidazole (Benz) and ribose abasic (Rab) **(c)** purine (P), 1-deazaadenosine (c^1A) and inosine (I) **(d)** 2,6-diamino (Dap) and 2-aminopurine-modified (Ap) codons was quantified (error bars show SDs from the mean of at least three independent experiments). Modified nucleotides are underlined and depicted in bold.

Supplementary Figure 2

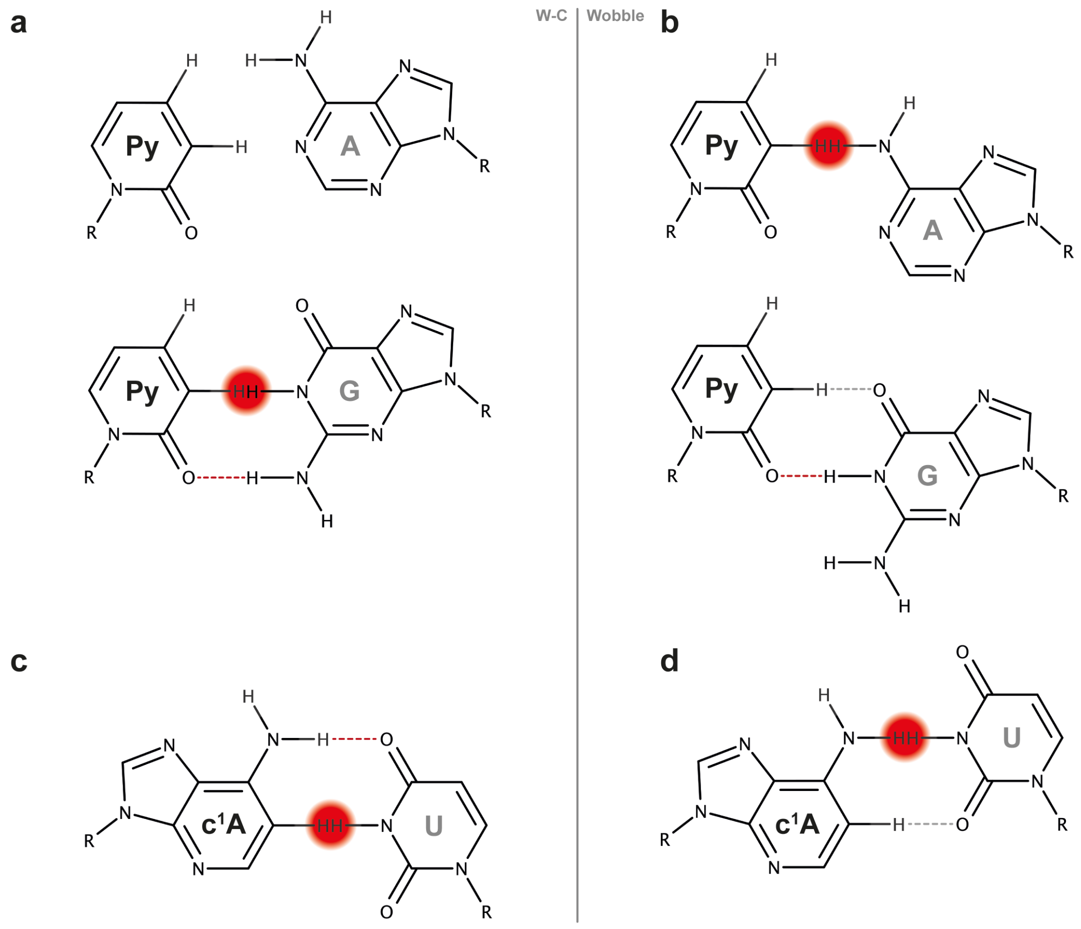

Supplementary Figure 2: Potential hydrogen bonds between pyridone (Py) and 1-deazaadenosine (c^{1}A) and standard bases. W-C base pair potential between **(a)** Py and A or G and between **(c)** c^{1}A and U are depicted. Wobble base pairs are drawn for **(b)** Py and A or G and for **(d)** c^{1}A and U. The H-bonds (red broken lines), C-H bonds (grey broken lines), and unfavorable contacts (red discs) are indicated.

Supplementary Figure 3

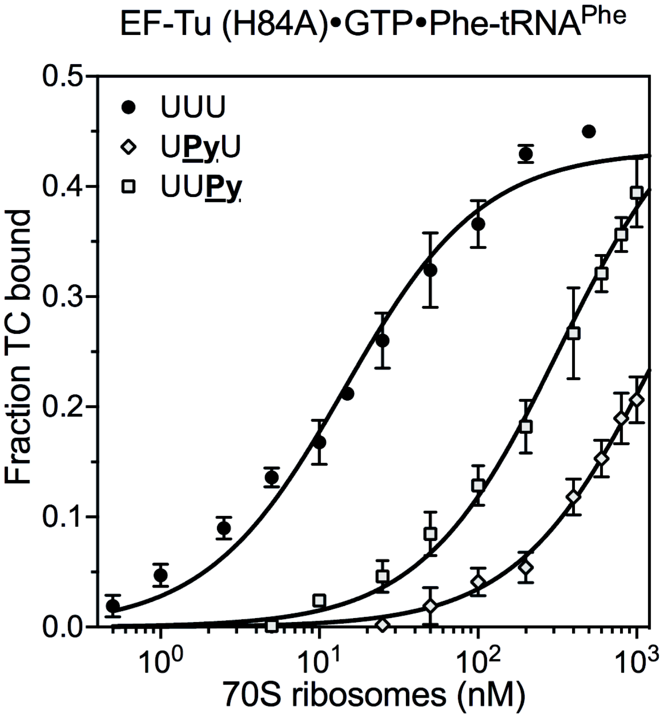

Supplementary Figure 3: Binding of EF-Tu (H84A)•GTP•Phe-tRNA^{Phe} to the 70S ribosome with pyridone-modified (Py) codons in the A site assessed by filter binding. Py reduced EF-Tu binding in a codon position-dependent manner. Binding of EF-Tu to PyUU in the A site was below the detection limit and is not displayed. Error bars depict the SDs from the mean of at least three independent experiments.

Supplementary Table 1: Thermodynamics of EF-Tu ternary complexes binding to the ribosome programmed with inosine- or pyridone-modified codons in the A site. Binding to PyUU was below the detection limit. K_D is depicted as K_D ± standard deviation from the mean of at least three independent experiments.

**A site codon**	**K_D (nM)**
GGG	49.1±2.0
**I**GG	205.3±8.9
G**I**G	127.9±7.8
GG**I**	64.8±3.5
UUU	14.4±1.3
**Py**UU	-
U**Py**U	1301±348
UU**Py**	325±40

Supplementary Figure 4

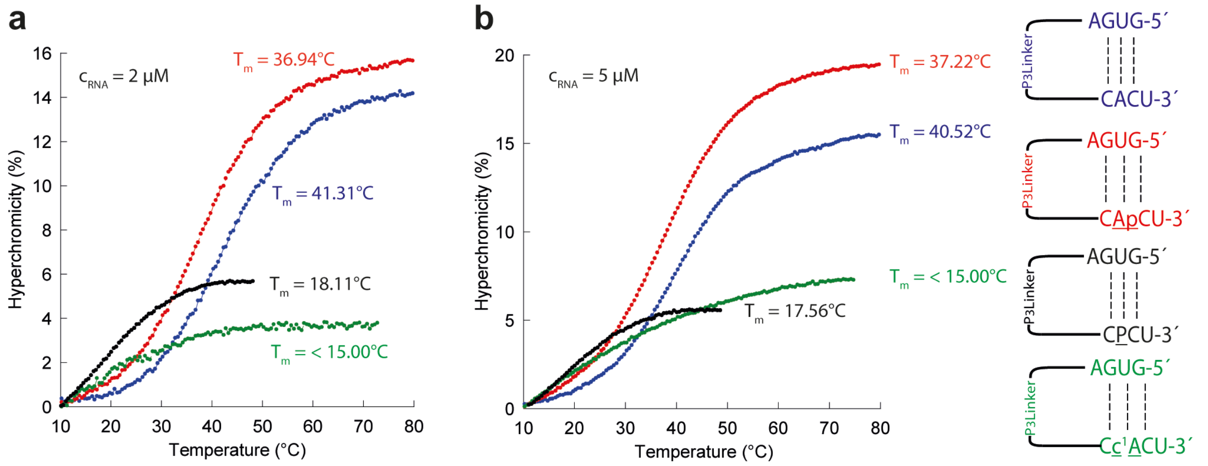

Supplementary Figure 4: The impact of single 2-aminopurines (AP), purines (P) and 1-deazaadenosines (c^1A) on the stability of the W-C interaction was investigated employing a minimal codon-anticodon model system. The hyperchromicity was measured at two distinct RNA oligonucleotide concentrations, at **(a)** 2 µM and at **(b)** 5 µM (8 µM for c^1A). Thereby, Ap at the minimal codon interacting with U at the minimal anticodon is more stable in comparison to the P-U interaction. The T_m for c^1A was below the detection limit and was estimated to be <15°C.

Supplementary Figure 5

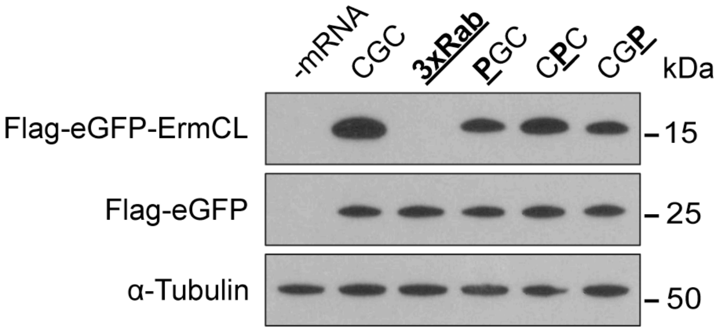

Supplementary Figure 5: Translation of purine-modified mRNAs in HEK293T cells. Purine was introduced in the eGFP-ErmCL mRNA and its translation was assessed by western blotting. An unmodified eGFP mRNA and α-tubulin served as an internal transfection control and as loading control, respectively.

Supplementary Table 2: MS analysis of the translation products isolated from the prokaryotic PURExpress *in vitro* translation system. Codons of interest are underlined and modified nucleotides are underlined and in bold. PSM, peptide spectrum match; XCorr, cross-correlation score.

mRNA Sequence	Peptides	Area	MH+ [Da]	# PSMs	XCorr
5'-[...] UAU **Ze**UU CCA AAC AAA AAA UAA-3'	mGMFSIFVISTVHYLPNKK	4,799E7	2240,16776	1	5,81
	FSIFVISTVHYLPNKK	2,377E9	1893,06936	1	5,51
	SIFVISTVHYLPNKK	3,706E8	1745,99370	3	4,64
	IFVISTVHYLPNKK	1,333E9	1658,96660	3	3,43
	SIFVISTVHYLPNK	1,512E8	1617,90569	2	4,35
	FVISTVHYLPNKK	1,069E9	1545,88469	3	4,35
	IFVISTVHYLPNK	1,489E8	1530,87126	2	3,99
	SIFVISTVHYLPN	2,183E8	1489,81255	1	3,43
	FVISTVHYLPNK	1,897E8	1417,78911	2	2,96
	IFVISTVHYLPN	9,118E7	1402,77690	1	2,45
	VISTVHYLPNKK	3,169E8	1398,81340	2	4,19
	ISTVHYLPNKK	2,877E10	1299,74504	11	3,36
	STVHYLPNKK	4,100E10	1186,66081	8	3,32
	ISTVHYLPNK	2,321E9	1171,64910	2	2,92
	TVHYLPNKK	1,430E9	1099,62529	2	2,78
	STVHYLPNK	4,444E9	1058,56523	3	3,25
	ISTVHYLPN	6,887E8	1043,55449	1	2,60
	VHYLPNKK	7,698E9	998,58031	4	2,51
	TVHYLPNK	1,097E9	971,53295	2	3,04
	HYLPNKK	1,040E10	899,51122	5	2,49
	HYLPNK	2,931E10	771,41612	1	2,53
5'-[...] UAU U**Ze**U CCA AAC AAA AAA UAA-3'	FSIFVISTVHYSPNKK	1,233E9	1867,01442	2	4,98
	SIFVISTVHYSPNKK	4,259E8	1719,94609	3	4,51
	IFVISTVHYSPNKK	7,664E8	1632,91472	4	4,64
	SIFVISTVHYSPNK	1,352E8	1591,85271	1	4,32
	FVISTVHYSPNKK	3,922E9	1519,83135	4	4,32
	IFVISTVHYSPNK	6,472E8	1504,81755	3	4,10
	SIFVISTVHYSPN	5,757E8	1463,75774	3	3,29
	IFVISTVHYSPN	5,520E8	1376,72392	1	2,60
	VISTVHYSPNKK	3,768E9	1372,76274	3	4,15
	STVHYSPNKK	1,917E9	1160,60758	3	4,06
	ISTVHYSPNK	2,115E9	1145,59807	3	3,35
	TVHYSPNKK	2,378E7	1073,57366	1	2,91
	STVHYSPNK	1,491E9	1032,51323	4	3,46
	ISTVHYSPN	1,238E9	1017,50188	2	2,90
	TVHYSPNK	9,824E7	945,48070	1	2,83
	ISTVHYFPNKK	5,894E8	1333,72783	1	3,74
	STVHYFPNKK	1,144E9	1220,64446	4	2,88
5'-[...] UAU **P**AA CCA AAC AAA AAA UAA-3'	mGmFSIFVISTVHYKPNKK	4,850E7	2271,164205	1	3,23
	mGMFSIFVISTVHYKPNKK	2,311E8	2255,162496	3	4,51
	mFSIFVISTVHYKPNKK	7,319E7	2067,105625	2	3,79
	FSIFVISTVHYKPNKK	3,013E8	1908,071934	3	4,40
	FSIFVISTVHYKPNK	2,683E7	1779,976475	1	2,99
	SIFVISTVHYKPNKK	1,859E8	1761,003818	2	4,28
	SIFVISTVHYKPNK	1,224E7	1632,909092	1	2,64
	IFVISTVHYKPNKK	3,030E9	1673,971226	4	4,00
	IFVISTVHYKPNK	6,933E7	1545,874899	1	3,53
	FVISTVHYKPNKK	1,242E9	1560,887485	2	3,07
	VISTVHYKPNKK	1,404E9	1413,818746	6	3,13
	VISTVHYKPNK	2,931E7	1285,724094	1	2,56

	ISTVHYKPNKK	1,103E10	1314,750143	6	3,54
	ISTVHYKPNK	5,746E8	1186,656298	2	3,53
	STVHYKPNKK	1,900E8	1201,66486	4	3,15
5'-[...] UAU APA CCA AAC AAA AAA UAA-3'	mGmFSIFVISTVHYKPNKK	3,362E7	2271,16445	1	2,50
	mGMFSIFVISTVHYKPNKK	1,625E8	2255,17069	4	4,93
	FSIFVISTVHYKPNKK	3,191E8	1908,06754	4	4,04
	FSIFVISTVHYKPNK	2,791E7	1779,97409	1	3,68
	SIFVISTVHYKPNKK	3,367E8	1761,00565	3	4,45
	SIFVISTVHYKPNK	2,531E7	1632,91220	1	2,72
	IFVISTVHYKPNKK	4,985E9	1673,97361	7	4,71
	IFVISTVHYKPNK	1,205E8	1545,87687	4	2,76
	IFVISTVHYKPN	6,625E7	1417,78315	1	2,37
	FVISTVHYKPNKK	1,725E9	1560,88913	5	3,74
	FVISTVHYKPNK	3,803E7	1432,79560	1	2,36
	VISTVHYKPNK	4,115E7	1285,72390	1	3,34
	VISTVHYKPNKK	1,873E9	1413,81923	5	3,30
	ISTVHYKPNKK	1,873E10	1314,75051	12	3,42
	ISTVHYKPNK	7,612E8	1186,65569	5	2,64
	STVHYKPNKK	3,436E8	1201,66596	6	3,38
5'-[...] UAU IGG CCA AAC AAA AAA UAA-3'	mGmFSIFVISTVHYGPNKK	6,299E8	2200,09897	5	6,14
	mGMFSIFVISTVHYGPNKK	4,215E8	2184,10874	2	5,90
	MGMFSIFVISTVHYGPNKK	4,111E8	2156,11063	3	6,22
	mFSIFVISTVHYGPNKK	4,580E8	1996,04038	1	5,25
	SIFVISTVHYGPNKK	3,359E9	1689,93559	4	3,89
	IFVISTVHYGPNKK	4,238E9	1602,90666	6	3,95
	SIFVISTVHYGPNK	5,003E8	1561,84538	2	3,70
	FVISTVHYGPNKK	4,778E9	1489,81767	5	4,15
	SIFVISTVHYGPN	5,956E8	1433,74468	3	3,85
	FVISTVHYGPNK	5,365E8	1361,72258	1	2,46
	IFVISTVHYGPN	2,024E8	1346,71379	1	2,62
	VISTVHYGPNKK	1,094E9	1342,74993	3	3,55
	ISTVHYGPNKK	5,269E10	1243,68108	14	3,65
	ISTVHYGPNK	3,862E9	1115,58525	4	3,29
	STVHYGPNK	1,287E9	1002,50163	3	3,53
	ISTVHYGPN	2,839E9	987,49040	1	2,63
	STVHYGPN	6,790E9	874,40679	5	3,19
	ISTVHYQPNKK	4,320E8	1314,71855	2	2,74
	ISTVHYQPNK	3,329E8	1186,62419	1	2,29
	ISTVHYQPN	4,085E8	1058,52812	2	2,78
5'-[...] UAU GIG CCA AAC AAA AAA UAA-3'	mGmFSIFVISTVHYGPNKK	6,228E8	2200,09366	6	6,12
	mGMFSIFVISTVHYGPNKK	8,805E9	2184,10434	2	6,00
	MGMFSIFVISTVHYGPNKK	1,495E9	2156,10587	3	6,13
	mFSIFVISTVHYGPNKK	8,572E8	1996,03653	1	5,26
	FSIFVISTVHYGPNKK	1,709E9	1837,00161	2	5,53
	SIFVISTVHYGPNKK	2,847E9	1689,93706	4	4,07
	IFVISTVHYGPNKK	2,236E9	1602,90349	6	4,08
	SIFVISTVHYGPNK	1,462E8	1561,83794	1	3,82
	FVISTVHYGPNKK	3,965E9	1489,81914	6	4,20
	SIFVISTVHYGPN	2,683E8	1433,74419	2	3,56
	FVISTVHYGPNK	2,865E8	1361,72246	1	2,76
	VISTVHYGPNKK	3,011E8	1342,74944	2	3,82
	ISTVHYGPNKK	5,510E9	1243,68022	8	3,70
	STVHYGPNKK	4,068E8	1130,59587	5	3,42
	ISTVHYGPNK	4,572E8	1115,58550	2	3,33
	STVHYGPNK	1,622E8	1002,50084	4	3,57
	ISTVHYGPN	4,662E8	987,49108	1	2,69
	STVHYGPN	3,842E8	874,40611	2	3,34

	mGmFSIFVISTVHYVPNKK	1,640E8	2242,14292	1	5,73
	IFVISTVHYVPNKK	2,505E8	1644,95134	2	4,50
	FVISTVHYVPNKK	2,267E8	1531,86601	2	4,52
	VISTVHYVPNKK	3,455E7	1384,79375	1	3,92
	ISTVHYVPNKK	1,866E9	1285,72795	3	4,07
	STVHYVPNKK	6,937E8	1172,64311	3	3,45
	TVHYVPNKK	2,756E7	1085,61260	1	2,45
5'-[...] UAU **Dap**GG CCA AAC AAA AAA UAA-3'	mGmFSIFVISTVHYRPNKK	6,640E8	2299,18357	7	5,86
	mFSIFVISTVHYRPNKK	1,744E9	2095,11948	2	5,21
	FSIFVISTVHYRPNKK	6,085E8	1936,08535	2	5,32
	SIFVISTVHYRPNKK	1,726E8	1789,02019	1	3,72
	IFVISTVHYRPNKK	7,099E8	1701,98552	5	3,85
	SIFVISTVHYRPNK	7,298E7	1660,92668	1	3,99
	FVISTVHYRPNKK	7,549E8	1588,89934	3	3,93
	IFVISTVHYRPNK	7,754E7	1573,89348	1	3,83
	FVISTVHYRPNK	2,002E8	1460,80535	1	3,74
	VISTVHYRPNKK	1,773E8	1441,83232	2	3,30
	ISTVHYRPNKK	7,389E9	1342,76225	11	3,61
	FVISTVHYRPN	1,792E8	1332,70915	1	3,39
	ISTVHYRPNK	2,458E9	1214,66789	2	2,87
	ISTVHYRPN	1,260E9	1086,57097	1	2,76
5'-[...] UAU G**Dap**G CCA AAC AAA AAA UAA-3'	mGmFSIFVISTVHYEPNKK	1,326E9	2272,12204	6	6,36
	mGMFSIFVISTVHYEPNKK	2,415E9	2256,13272	5	5,96
	MGMFSIFVISTVHYEPNKK	1,696E9	2228,12656	7	6,72
	MFSIFVISTVHYEPNKK	5,098E8	2040,06351	1	5,38
	FSIFVISTVHYEPNKK	1,461E8	1909,02800	1	5,44
	SIFVISTVHYEPNKK	1,323E9	1761,95964	5	4,73
	IFVISTVHYEPNKK	1,724E9	1674,92620	6	4,50
	FVISTVHYEPNKK	4,148E9	1561,84331	4	4,86
	IFVISTVHYEPNK	1,673E8	1546,82915	1	3,76
	SIFVISTVHYEPN	1,234E9	1505,76921	4	3,96
	FVISTVHYEPNK	2,483E8	1433,74529	1	3,41
	IFVISTVHYEPN	8,388E8	1418,73491	2	2,95
	VISTVHYEPNKK	8,220E8	1414,77373	3	3,86
	SIFVISTVHYEP	1,804E8	1391,72331	1	3,05
	ISTVHYEPNKK	2,565E10	1315,70403	10	4,10
	STVHYEPNKK	1,705E10	1202,62029	12	3,62
	ISTVHYEPNK	1,120E9	1187,60747	2	3,01
	TVHYEPNKK	4,123E7	1115,59026	2	2,89
	STVHYEPNK	2,085E9	1074,52471	3	3,09
	ISTVHYEPN	6,067E9	1059,51445	1	2,82
5'-[...] UAU **Ap**GG CCA AAC AAA AAA UAA-3'	mGmFSIFVISTVHYRPNKK	2,266E8	2299,17514	1	5,63
	mFSIFVISTVHYRPNKK	1,350E7	2095,12882	2	4,92
	FSIFVISTVHYRPNKK	2,904E8	1936,08291	2	5,06
	IFVISTVHYRPNKK	1,905E8	1701,97600	1	3,87
	FVISTVHYRPNKK	6,198E8	1588,89775	3	4,12
	SIFVISTVHYRPN	1,282E8	1532,83550	1	3,30
	FVISTVHYRPNK	6,667E7	1460,80571	1	3,41
	IFVISTVHYRPN	5,322E8	1445,79489	1	4,93
	VISTVHYRPNKK	1,443E8	1441,83220	1	3,27
	ISTVHYRPNKK	6,874E9	1342,76164	10	3,58
	FVISTVHYRPN	1,720E8	1332,70745	1	2,70
	SIFVISTVHYR	1,387E8	1321,73015	1	2,77
	STVHYRPNKK	1,664E8	1229,67681	4	3,19
	ISTVHYRPNK	1,585E9	1214,66728	1	2,69
	ISTVHYRPN	1,206E9	1086,57170	1	2,76
	ISTVHYWPNKK	7,699E8	1372,74175	4	3,76

	STVHYWPNKK	7,453E8	1259,65679	1	2,69
	HYWPNKK	1,815E8	972,50737	1	2,63
5'-[...] UAU **GApG** CCA AAC AAA AAA UAA-3'	mGMFSIFVISTVHYEPNKK	1,467E8	2256,12613	1	6,40
	FSIFVISTVHYEPNKK	4,003E9	1909,02700	9	4,90
	SIFVISTVHYEPNKK	9,496E9	1761,95717	6	4,73
	IFVISTVHYEPNKK	4,687E10	1674,92630	7	4,28
	FVISTVHYEPNKK	2,584E9	1561,84319	5	4,09
	SIFVISTVHYEPN	1,693E9	1505,76775	4	3,70
	FVISTVHYEPNK	1,632E8	1433,75481	1	3,63
	IFVISTVHYEPN	1,566E9	1418,73467	1	2,67
	VISTVHYEPNKK	3,084E8	1414,77239	2	3,68
	ISTVHYEPNKK	1,702E10	1315,70378	8	3,56
	STVHYEPNKK	7,567E9	1202,61870	6	3,57
	ISTVHYEPNK	7,355E8	1187,60918	1	2,64
	STVHYEPNK	1,195E9	1074,52397	3	2,98
	ISTVHYEPN	6,500E9	1059,51152	1	2,68
	STVHYEPN	8,875E9	946,42894	1	2,54
	ISTVHYGPNKK	1,302E9	1243,68254	3	3,34
	STVHYGPNK	3,043E7	1002,50279	1	3,07
5'-[...] UAU AU**Rab** CCA AAC AAA AAA UAA-3'	SIFVISTVHYIPNKK	3,614E9	1745,99338	4	4,28
	IFVISTVHYIPNKK	9,158E9	1658,96189	7	4,01
	FVISTVHYIPNKK	5,703E8	1545,87212	1	4,38
	VISTVHYIPNKK	9,200E7	1398,81377	1	3,38
	ISTVHYIPNKK	6,902E10	1299,74236	8	3,17
	STVHYIPNKK	1,064E11	1186,65781	13	3,42
	ISTVHYIPNK	2,136E9	1171,64490	3	2,99
	TVHYIPNKK	5,005E9	1099,62375	11	3,20
	STVHYIPNK	1,184E9	1058,56304	1	2,89
	SIFVISTVH	1,836E9	1002,56096	3	2,49
	VHYIPNKK	3,564E8	998,57848	1	2,20
	TVHYIPNK	3,440E8	971,53099	1	2,37
	SIFVISTVHYMPNKK	1,065E9	1763,95127	1	3,83
	IFVISTVHYMPNKK	2,117E9	1676,91776	3	4,11
	FVISTVHYMPNKK	0,000E0	1563,83794	2	3,48
	ISTVHYMPNKK	4,591E9	1317,69853	3	2,58
	STVHYMPNKK	5,488E9	1204,61455	4	3,10
	ISTVHYMPNK	1,415E8	1189,60417	1	2,72
	TVHYMPNKK	8,727E7	1117,58293	1	2,71
	STVHYMPNK	4,301E8	1076,51970	3	3,19
5'-[...] UAU AU**Dap** CCA AAC AAA AAA UAA-3'	mGMFSIFVISTVHYIPNKK	3,093E6	2240,17234	1	6,07
	SIFVISTVHYIPNKK	4,055E8	1746,00261	2	4,79
	IFVISTVHYIPNKK	6,458E8	1658,96953	3	3,95
	FVISTVHYIPNKK	4,557E8	1545,88237	3	4,38
	IFVISTVHYIPNK	5,633E7	1530,87908	1	4,28
	VISTVHYIPNKK	2,522E8	1398,81816	2	4,18
	ISTVHYIPNKK	1,057E11	1299,74640	8	4,10
	STVHYIPNKK	2,875E10	1186,66252	4	3,07
	ISTVHYIPNK	2,198E9	1171,65080	1	3,24
	TVHYIPNKK	1,650E9	1099,62981	1	2,55
	STVHYIPNK	3,547E9	1058,56719	4	2,88
	VHYIPNKK	5,857E9	998,58470	2	2,62
	HYIPNKK	7,561E8	899,51195	3	2,53
	HYIPNK	6,193E9	771,41747	1	2,54
	FSIFVISTVHYMPNKK	3,728E8	1911,02909	1	4,09
	SIFVISTVHYMPNKK	2,666E8	1763,95818	1	5,02
	IFVISTVHYMPNKK	2,591E8	1676,92375	2	4,67
	FVISTVHYMPNKK	3,002E8	1563,83989	2	4,61

	SIFVISTVHYMPN	1,457E8	1507,76348	1	3,21
	VISTVHYMPNKK	1,569E8	1416,77153	1	3,89
	ISTVHYMPNKK	7,941E8	1333,69682	8	3,61
	ISTVHYMPNKK	1,322E10	1317,70317	7	3,45
	STVHYMPNKK	2,131E9	1220,61138	13	3,35
	STVHYMPNKK	2,013E10	1204,61968	8	3,51
	TVHYMPNKK	2,888E8	1133,58220	1	2,63
	STVHYMPNK	1,331E8	1092,51860	1	2,85
	STVHYMPNK	1,619E9	1076,52324	3	3,25
	mGMFSIFVISTVHYIPNKK	2,917E8	2240,16300	2	4,84
	SIFVISTVHYIPNKK	1,467E8	1745,99741	2	4,29
	FSIFVISTVHYIPNKK	8,244E7	1893,06534	2	3,95
	IFVISTVHYIPNKK	1,152E9	1658,96537	3	3,91
	mGmFSIFVISTVHYIPNKK	5,769E7	2256,15818	2	3,62
	mFSIFVISTVHYIPNKK	9,078E7	2052,09940	1	3,50
	STVHYIPNKK	7,640E9	1186,65625	7	3,44
	TVHYIPNKK	4,445E8	1099,62595	6	3,41
	ISTVHYIPNK	3,383E8	1171,64617	2	3,24
	ISTVHYIPNKK	3,396E9	1299,74614	5	3,05
	STVHYIPNK	2,655E8	1058,56235	1	2,93
	mGmFSIFVISTVHYMPNKK	2,930E8	2274,11368	7	5,86
	mGMFSIFVISTVHYMPNKK	1,345E9	2258,11869	4	5,71
	IFVISTVHYMPNKK	6,826E9	1676,91922	6	5,03
	SIFVISTVHYMPNKK	1,108E9	1763,95072	3	4,94
	FSIFVISTVHYMPNKK	1,058E9	1911,02140	4	4,23
5'-[...] UAU AU**Ap** CCA AAC AAA AAA UAA-3'	mFSIFVISTVHYMPNKK	5,448E8	2070,05619	3	4,01
	FVISTVHYMPNKK	2,380E9	1563,83818	3	3,61
	IFVISTVHYMPNKK	4,947E8	1692,91532	2	3,60
	STVHYMPNKK	3,459E10	1204,61451	13	3,49
	VISTVHYMPNKK	1,473E8	1416,76787	3	3,47
	FVISTVHYMPNK	8,502E7	1435,73883	1	3,46
	STVHYMPNK	1,067E9	1076,52129	4	3,34
	FSIFVISTVHYMPNK	6,727E7	1782,92209	1	3,27
	FSIFVISTVHYMPNKK	7,202E7	1927,01511	1	3,18
	TVHYMPNKK	2,381E9	1117,58219	11	3,10
	ISTVHYMPNK	2,090E9	1189,60552	4	2,98
	ISTVHYMPNKK	2,074E9	1317,69927	3	2,95
	STVHYMPNKK	1,528E9	1220,60749	2	2,80
	IFVISTVHYMPNK	2,854E8	1548,82651	3	2,79
	ISTVHYMPNKK	1,354E8	1333,69548	1	2,73
	VHYMPNKK	1,174E10	1016,53470	1	2,64
	TVHYMPNK	2,403E8	989,48857	1	2,28
	mGMFSIFVISTVHYIPNKK	1,466E8	2240,16245	2	4,71
	IFVISTVHYIPNKK	5,891E9	1658,96482	5	4,14
	SIFVISTVHYIPNKK	7,200E8	1745,99759	3	4,04
	FSIFVISTVHYIPNKK	4,088E8	1893,06571	4	3,81
	ISTVHYIPNK	1,954E9	1171,64800	5	3,53
	TVHYIPNKK	1,317E9	1099,62604	13	3,39
	STVHYIPNK	1,009E9	1058,56336	6	3,27
	STVHYIPNKK	2,228E10	1186,65898	16	3,26
	VISTVHYIPNKK	7,211E7	1398,81658	2	3,20
	ISTVHYIPNKK	1,908E10	1299,74221	11	3,01
	FVISTVHYIPNKK	1,781E9	1545,87868	3	3,00
	mGmFSIFVISTVHYIPNKK	4,016E7	2256,15927	2	2,95
5'-[...] UAU AU**P** CCA AAC AAA AAA UAA-3'	TVHYIPNK	6,495E8	971,53205	4	2,83
	VHYIPNKK	6,531E9	998,57892	12	2,63
	HYIPNK	1,082E9	771,41478	8	2,55

	FSIFVISTVHYIPNK	1,827E7	1764,97080	1	2,55
	mFSIFVISTVHYIPNKK	6,959E7	2052,09958	1	2,52
	IFVISTVHYIPNK	1,755E8	1530,86798	1	2,36
	FVISTVHYIPNK	7,157E7	1417,78736	1	2,35
	HYIPNKK	2,598E8	899,50981	2	2,31
	VHYIPNK	1,289E8	870,48430	1	2,06
	STVHYMPNKK	4,491E8	1204,61377	4	3,21
	SIFVISTVHYMPNKK	1,712E7	1763,95420	1	2,88
	ISTVHYMPNKK	5,074E8	1317,69953	3	2,68
	VHYMPNKK	4,135E7	1016,53489	2	2,55
5'-[...] UAU AU**c1A** CCA AAC AAA AAA UAA-3'	mGMFSIFVISTVHYIPNKK	1,076E7	2240,16080	1	6,12
	mGmFSIFVISTVHYIPNKK	7,141E6	2256,15873	3	3,58
	IFVISTVHYIPNKK	4,956E6	1658,96079	8	3,42
	SIFVISTVHYIPNKK	3,733E6	1745,99887	6	3,36
	ISTVHYIPNKK	9,256E7	1299,74370	64	3,31
	TVHYIPNKK	4,181E7	1099,62650	13	3,02
	VHYIPNKK	2,150E7	998,57764	24	2,68
	VISTVHYIPNKK	6,042E7	1398,81388	39	2,59
	FVISTVHYIPNKK	2,834E7	1545,87944	27	2,46
	STVHYIPNKK	1,486E7	1186,65923	29	2,42
5'-[...] UAU AU**I** CCA AAC AAA AAA UAA-3'	ISTVHYIPNKK	2,595E8	1299,74675	2	3,07
	STVHYIPNKK	4,592E8	1186,66252	3	2,64
	mGMFSIFVISTVHYMPNKK	2,249E7	2258,12528	1	5,71
	MFSIFVISTVHYMPNKK	2,007E8	2042,06704	1	4,42
	FSIFVISTVHYMPNKK	1,339E9	1911,02724	3	4,88
	SIFVISTVHYMPNKK	5,927E9	1763,95717	4	4,22
	IFVISTVHYMPNKK	1,529E10	1676,92470	4	4,37
	SIFVISTVHYMPNK	1,048E8	1635,86345	1	4,23
	FVISTVHYMPNKK	9,120E9	1563,84024	4	4,06
	IFVISTVHYMPNK	7,760E7	1548,83147	1	3,61
	FVISTVHYMPNK	8,561E7	1435,74651	1	3,41
	VISTVHYMPNKK	1,033E9	1416,77446	3	4,23
	ISTVHYMPNKK	5,907E8	1333,69682	10	3,77
	ISTVHYMPNKK	2,158E10	1317,70293	12	3,24
	STVHYMPNKK	1,366E9	1220,61113	23	3,34
	STVHYMPNKK	3,062E10	1204,61872	15	3,97
	ISTVHYMPNK	3,557E9	1189,60723	2	2,49
	TVHYMPNKK	1,891E8	1133,58318	2	2,78
	TVHYMPNKK	2,900E9	1117,58623	1	2,58
	STVHYMPNK	1,695E8	1092,51909	3	3,37
	STVHYMPNK	3,777E9	1076,52373	3	2,80
	ISTVHYMPN	9,142E8	1061,51225	1	2,39
	VHYMPNKK	4,334E8	1032,53301	3	2,64
	VHYMPNKK	5,597E9	1016,53856	4	2,55
	HYMPNKK	9,768E7	917,46916	2	2,52
	VHYMPNK	4,970E8	888,44255	1	2,53
	HYMPNK	4,026E8	805,36833	6	2,46
	HYMPNK	5,990E9	789,37383	2	2,44

Supplementary Table 3: MS analysis of the translation products isolated from HEK293T cells 24 h after transfection. Codons of interest are underlined and modified nucleotides are underlined and in bold. PSM, peptide spectrum match; XCorr, cross-correlation score.

mRNA sequence	Peptides	Area	MH+ [Da]	# PSMs	XCorr
5'-[...] UAU **Ze**UU CCA AAC AAA AAA UAA-3'	GIDFKEDDYLPNKK	4,50E+09	1681,84318	42	4,50
	GIDFKEDDYLPNK	2,15E+07	1553,74821	2	2,75
	EDDYLPNKK	9,71E+06	1121,54733	4	1,74
	DFKEDDYLPNKK	3,41E+07	1511,73765	2	1,92
5'-[...] UAU U**Ze**U CCA AAC AAA AAA UAA-3'	GIDFKEDDYSPNKK	4,61E+09	1655,79114	18	4,50
	GIDFKEDDYSPNK	9,66E+07	1527,69618	3	3,06
	IDFKEDDYSPNKK	6,96E+07	1598,76968	2	2,32
	DFKEDDYSPNKK	2,75E+07	1485,68561	1	2,83
	GIDFKEDDYFPNKK	7,93E+07	1715,82753	3	3,91
5'-[...] UAU **P**AA CCA AAC AAA AAA UAA-3'	GIDFKEDDYKPN	2,60E+07	1440,66415	1	1,94
	GIDFKEDDYKPNK	7,93E+07	1568,75911	4	2,91
	GIDFKEDDYKPNKK	3,95E+09	1696,85408	11	4,52
5'-[...] UAU A**P**A CCA AAC AAA AAA UAA-3'	GIDFKEDDYKPNKK	2,07E+08	1696,85408	7	2,79
5'-[...] UAU **I**GG CCA AAC AAA AAA UAA-3'	ELKGIDFKEDDYGPNKK	1,15E+07	1996,00220	1	2,55
	GIDFKEDDYGPNK	4,63E+07	1497,68561	2	1,86
	GIDFKEDDYGPNKK	3,11E+09	1625,78058	19	4,65
	GIDFKEDDYRPNKK	6,12E+07	1724,86022	2	2,24
5'-[...] UAU G**I**G CCA AAC AAA AAA UAA-3'	IELKGIDFKEDDYGPNKK	6,83E+07	2109,08626	2	2,75
	GIDFKEDDYGPNK	1,46E+08	1497,68561	4	2,64
	GIDFKEDDYGPNKK	7,06E+09	1625,78058	22	4,28
	IDFKEDDYGPNKK	2,93E+07	1568,75911	1	1,76
	DFKEDDYGPNKK	3,60E+07	1455,67505	1	3,30
5'-[...] UAU **Dap**GG CCA AAC AAA AAA UAA-3'	GIDFKEDDYRPNK	5,26E+07	1596,76526	1	2,69
	GIDFKEDDYRPNKK	2,45E+09	1724,86022	15	3,72
5'-[...] UAU G**Dap**G CCA AAC AAA AAA UAA-3'	IELKGIDFKEDDYEPNKK	3,30E+07	2181,10739	1	2,64
	GIDFKEDDYEPNK	7,93E+07	1569,70674	2	2,00
	GIDFKEDDYEPNKK	6,74E+09	1697,80171	21	4,20
	IDFKEDDYEPNKK	2,11E+07	1640,78024	2	2,18
	DFKEDDYEPNKK	2,84E+07	1527,69618	1	2,45
5'-[...] UAU **Ap**GG CCA AAC AAA AAA UAA-3'	GIDFKEDDYRPNK	3,52E+07	1596,76526	1	2,15
	GIDFKEDDYRPNKK	1,56E+09	1724,86022	13	4,07
5'-[...] UAU G**Ap**G CCA AAC AAA AAA UAA-3'	ELKGIDFKEDDYEPNKK	2,66E+07	2068,02333	1	2,69
	GIDFKEDDYEPNKK	1,83E+09	1697,80171	13	3,80
5'-[...] UAU AU**Dap** CCA AAC AAA AAA UAA-3'	EDDYIPNKK	2,35E+08	1121,54733	8	2,72
	GIDFKEDDYIPNK	8,37E+07	1553,74821	2	3,46
	GIDFKEDDYIPNKK	5,85E+09	1681,84318	48	4,27
	GIDFKEDDYMPNKK	1,31E+08	1699,79960	1	3,96
5'-[...] UAU AU**Ap** CCA AAC AAA AAA UAA-3'	GIDFKEDDYIPNKK	2,98E+09	1681,84318	26	4,01
	EDDYIPNKK	8,52E+07	1121,54733	6	1,96
	GIDFKEDDYMPNKK	9,36E+08	1699,79960	8	3,69
	GIDFKEDDYMPNKK	2,50E+08	1715,79451	4	4,20
5'-[...] UAU AU**P** CCA AAC AAA AAA UAA-3'	GIDFKEDDYIPNKK	1,70E+09	1681,84318	24	4,67
5'-[...] UAU AU**I** CCA AAC AAA AAA UAA-3'	GIDFKEDDYMPNKK	3,37E+09	1699,79960	11	4,63
	GIDFKEDDYMPNKK	7,52E+08	1715,79451	7	3,22
	GIDFKEDDYMPNK	6,03E+07	1571,70463	1	1,60
	TRAEVKFEGDTLVNRIELK GIDFKEDVAIRSPIEHS	3,79E+09	4112,17748	15	8,91

5'-[...] GAC GUA GCA AUA CGU <u>A**I**U</u> CCU AUU GAG CAU AGC UAA-3' (5-HT_{2C})	**FEGDTLVNRIELK GIDFKEDVAIRSPIEHS**	1,15E+09	3427,78561	6	7,69
	**GIDFKEDVAIRSPIEHS**	2,72E+07	1912,97632	4	5,11
	**EDVAIRSPIEHS**	6,87E+07	1352,68047	3	2,25
5'-[...] AAG GAG GAC GAU AUG <u>C**I**G</u> CAA AAC AAA AAA UAA-3' (GluR-B)	**TRAEVKFEGDTLVNRIELK GIDFKEDDMRQNKK**	1,40E+09	3923,04436	11	8,75
	**TRAEVKFEGDTLVNRIELK GIDFKEDDMRQNKK**	3,39E+08	3939,03927	5	8,32
	**TRAEVKFEGDTLVNRIELK GIDFKEDDMRQNK**	3,16E+07	3794,94940	1	3,09
	**FEGDTLVNRIELK GIDFKEDDMRQNKK**	1,76E+07	3254,64740	1	2,88
	**FEGDTLVNRIELK GIDFKEDDMRQNKK**	2,34E+08	3238,65249	7	6,47
	**GIDFKEDDMRQNKK**	1,57E+07	1723,84319	3	1,61

Supplementary Table 4: Overview of translation fidelity and accuracy of modified codons in prokaryotes and eukaryotes (n.d., not determined). The translation efficiency was normalized to the translation of the respective unmodified mRNA (Mean ± standard deviation of at least three independent experiments is depicted).

		Decoding	
**Codon**	***E. coli* IVT efficiency**	**Prokaryotes**	**Eukaryotes**
**Ze**UU	113%±12%	100% Leu	100% Leu
U**Ze**U	95%±14%	91.6% Ser, 8.4% Phe	98.3% Ser, 1.7% Phe
UU**Ze**	159%±10%	n.d.	n.d.
**Py**UU	4%±6%	n.d.	n.d.
U**Py**U	4%±4%	n.d.	n.d.
UU**Py**	99%±5%	n.d.	n.d.
**Benz**GG	3%±3%	n.d.	n.d.
G**Benz**G	1%±1%	n.d.	n.d.
GG**Benz**	67%±8%	n.d.	n.d.
**Rab**GG	1%±1%	n.d.	n.d.
G**Rab**G	2%±0%	n.d.	n.d.
GG**Rab**	3%±1%	n.d.	n.d.
**P**AA	76%±14%	100% Lys	100% Lys
A**P**A	75%±10%	100% Lys	100% Lys
AA**P**	85%±5%	n.d.	n.d.
**c^1A**AA	2%	n.d.	n.d.
A**c^1A**A	1%	n.d.	n.d.
AA**c^1A**	1%	n.d.	n.d.
**I**GG	71%±11%	98.2%, 1.4% Gln, 0.4% Arg	98.1% Gly, 1.9% Arg
G**I**G	93%±10%	90.5% Gly, 9.5% Val	100% Gly
GG**I**	92%±11%	n.d.	n.d.
**Dap**GG	65%±7%	100% Arg	100% Arg
G**Dap**G	82%±9%	100% Glu	100% Glu
GG**Dap**	84%±16%	n.d.	n.d.
**Ap**GG	59%±13%	87.9% Arg, 12.1% Trp	100% Arg
G**Ap**G	57%±12%	98.8% Glu, 1.2% Gly	100% Glu
GG**Ap**	86%±7%	n.d.	n.d.
AU**Rab**	n.d.	93.5% Ile, 6.5% Met	n.d.
AU**Dap**	n.d.	79.7% Ile, 20.3% Met	97.8% Ile, 2.2% Met
AU**Ap**	n.d.	83.5% Met, 16.5% Ile	71.5% Ile, 28.5% Met
AU**P**	n.d.	98.3% Ile, 1.7% Met	100% Ile
AU**c^1A**	n.d.	100% Ile	n.d.
AU**I**	n.d.	99.4% Met, 0.6% Ile	100% Met

Supplementary Figure 6

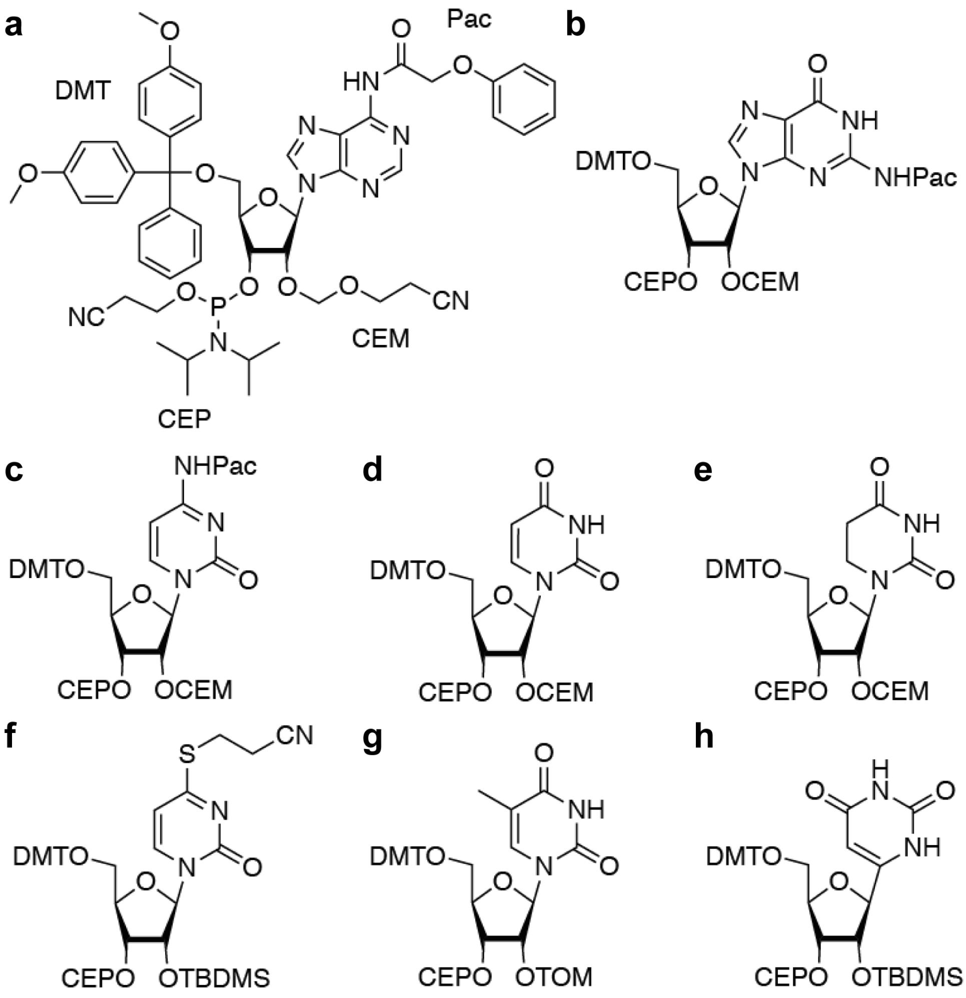

Supplementary Figure 6: **Used phosphoramidites for the synthesis of *E. coli* tRNA^{Gly}. (a)** N^{6}-Phenoxyacetyl(Pac)-2'-Cyanoethoxymethyl(CEM)-3`-(2-cyanoethy-N,N-diisopropylaminophosphoramidite)(CEP)-5'4,4'-dimethoxytrityl(DMT)-adenosine; **(b)** N^{2}-Pac-2'CEM-3'CEP-5'DMT-guanosine; **(c)** N^{4}-Pac-2'CEM-3'CEP-5'DMT-cytidine; **(d)** 2'CEM-3'CEP-5'DMT-uridine; **(e)** 2'CEM-3'CEP-5'DMT-dihydrouridine; **(f)** S^{4}-CE-2'Tertbutyldimethylsilyl(TBDMS)-3'CEP-5'DMT-thiouridine **(g)** 2'[(triisopropylsilyl)oxy]methyl(TOM)-3'CEP-5'DMT-5-methyl-uridine; **(h)** 2'TBDMS-3'CEP-5'DMT-pseudouridine.

Supplementary Figure 7

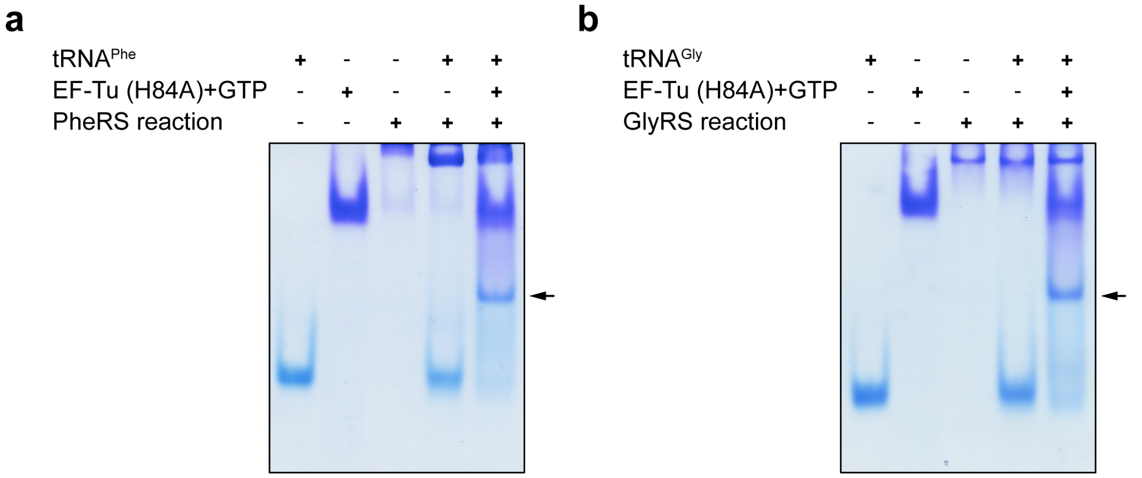

Supplementary Figure 7: EF-Tu activity and ternary complex formation assessed by native gels. A 3-fold excess of EF-Tu (H84A) over **(a)** Phe-tRNA^{Phe} or **(b)** Gly-tRNA^{Gly} leads to an almost quantitative ternary complex formation (arrow). Gels were stained with Coomassie Blue (purple) and Methylene Blue (light blue) to visualize proteins and tRNAs, respectively.

Supplementary Figure 8

Figure 2B

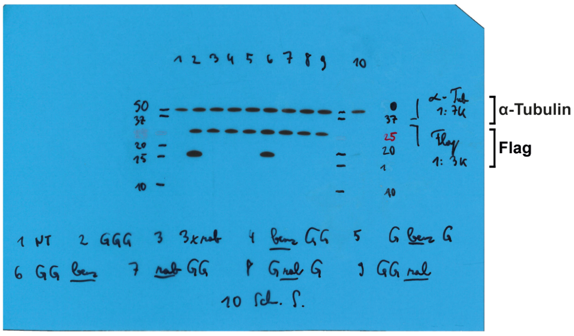

Figure 2D

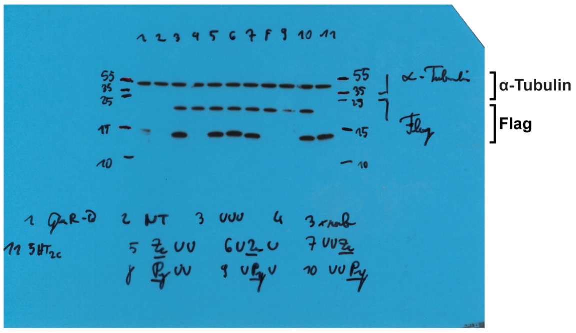

Figure 2F

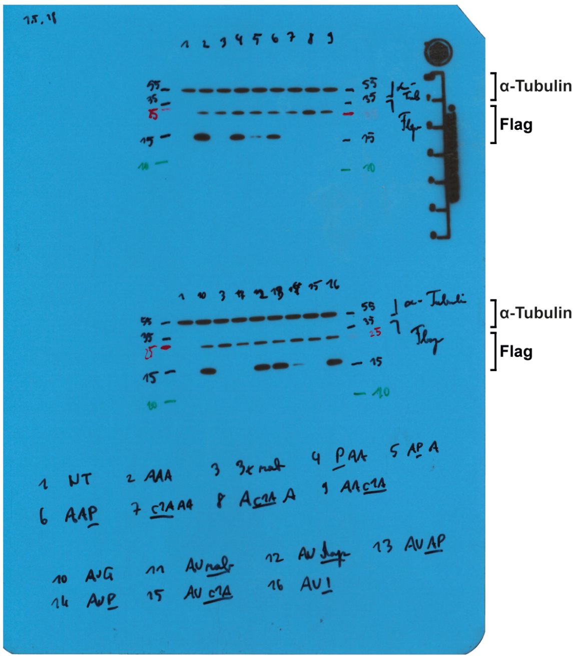

Figure 2H

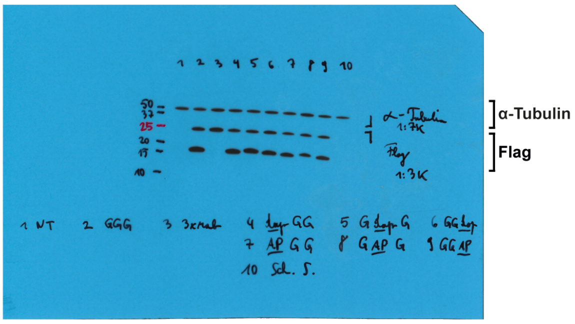

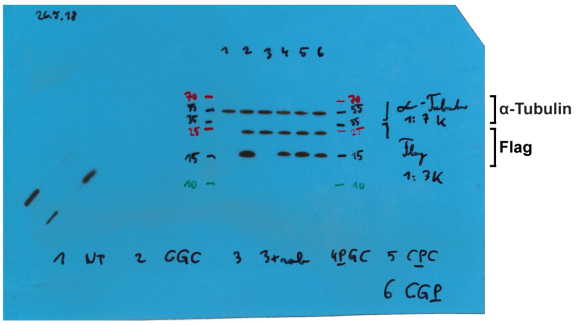

Figure 3B

Supplementary Figure 5

Figure 4D

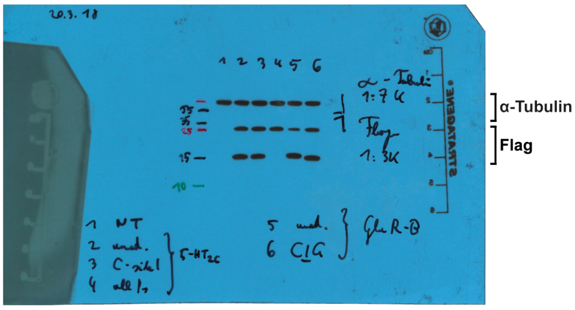

Figure 5F

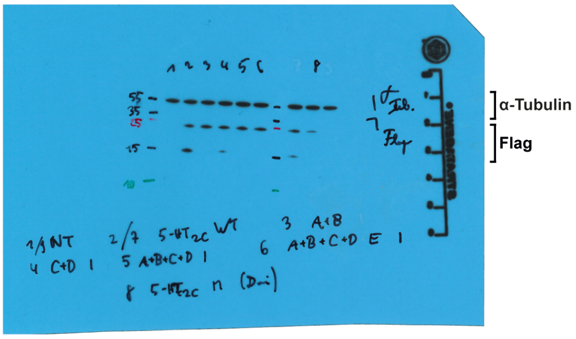

Supplementary Figure 8: Uncropped scans of the western blots displayed in various main and supplemental figures. Arrows indicate the lanes depicted in the respective figures.
