# Supplementary material for: Translation of non-standard codon nucleotides reveals minimal requirements for codon-anticodon interactions
Source: Nat Commun. 2018 Nov 19;9:4865. doi: 10.1038/s41467-018-07321-8 (PMC6242847; doi:10.1038/s41467-018-07321-8)
Supplement: Supplementary file 3 — Reporting Summary [file 41467_2018_7321_MOESM3_ESM.pdf]

Corresponding author(s): Matthias Erlacher



# Reporting Summary

Nature Research wishes to improve the reproducibility of the work that we publish. This form provides structure for consistency and transparency in reporting. For further information on Nature Research policies, see Authors & Referees and the Editorial Policy Checklist.

## Statistical parameters

When statistical analyses are reported, confirm that the following items are present in the relevant location (e.g. figure legend, table legend, main text, or Methods section).

| n/a | Confirmed | |
|---|---|---|
| ☒ | ☐ | The exact sample size (*n*) for each experimental group/condition, given as a discrete number and unit of measurement |
| ☐ | ☒ | An indication of whether measurements were taken from distinct samples or whether the same sample was measured repeatedly |
| ☒ | ☐ | The statistical test(s) used AND whether they are one- or two-sided<br>*Only common tests should be described solely by name; describe more complex techniques in the Methods section.* |
| ☒ | ☐ | A description of all covariates tested |
| ☒ | ☐ | A description of any assumptions or corrections, such as tests of normality and adjustment for multiple comparisons |
| ☐ | ☒ | A full description of the statistics including central tendency (e.g. means) or other basic estimates (e.g. regression coefficient) AND variation (e.g. standard deviation) or associated estimates of uncertainty (e.g. confidence intervals) |
| ☒ | ☐ | For null hypothesis testing, the test statistic (e.g. *F*, *t*, *r*) with confidence intervals, effect sizes, degrees of freedom and *P* value noted<br>*Give P values as exact values whenever suitable.* |
| ☒ | ☐ | For Bayesian analysis, information on the choice of priors and Markov chain Monte Carlo settings |
| ☒ | ☐ | For hierarchical and complex designs, identification of the appropriate level for tests and full reporting of outcomes |
| ☒ | ☐ | Estimates of effect sizes (e.g. Cohen's *d*, Pearson's *r*), indicating how they were calculated |
| ☐ | ☒ | Clearly defined error bars<br>*State explicitly what error bars represent (e.g. SD, SE, CI)* |

*Our web collection on statistics for biologists may be useful.*

## Software and code

Policy information about availability of computer code

| | |
|---|---|
| Data collection | No particular data collection software was used. |
| Data analysis | Data analyses were performed by GraphPad Prism 7 (7.0a, Mac). |

For manuscripts utilizing custom algorithms or software that are central to the research but not yet described in published literature, software must be made available to editors/reviewers upon request. We strongly encourage code deposition in a community repository (e.g. GitHub). See the Nature Research guidelines for submitting code & software for further information.

## Data

Policy information about availability of data

All manuscripts must include a data availability statement. This statement should provide the following information, where applicable:

- Accession codes, unique identifiers, or web links for publicly available datasets
- A list of figures that have associated raw data
- A description of any restrictions on data availability

All mass spectrometry data have been deposited to the ProteomeXchange Consortium via the PRIDE database with the data set identifiers PXD011311 (E. coli PURExpress translation assay) and PXD011301 (translation in HEK293T cells). All other data supporting the findings of this study are available within this article and in the Supplementary Information or from the corresponding author upon reasonable request.

April 2018

# Field-specific reporting

Please select the best fit for your research. If you are not sure, read the appropriate sections before making your selection.

☒ Life sciences ☐ Behavioural & social sciences ☐ Ecological, evolutionary & environmental sciences

For a reference copy of the document with all sections, see nature.com/authors/policies/ReportingSummary-flat.pdf

# Life sciences study design

All studies must disclose on these points even when the disclosure is negative.

| | |
|---|---|
| Sample size | Sample-size calculations were not required in the case of this study. Experiments were independently repeated at least three times as indicated and mean and the standard deviation from the mean were calculated. |
| Data exclusions | Experiments were excluded that were technically invalid (e.g., EF-TU binding below biological relevant level as an indicator). |
| Replication | The replication of all experiments was successful. |
| Randomization | Randomization was not relevant for this study. |
| Blinding | Blinding was not relevant for this study. |

# Reporting for specific materials, systems and methods

## Materials & experimental systems

| n/a | Involved in the study |
|---|---|
| ☒ | ☐ Unique biological materials |
| ☐ | ☒ Antibodies |
| ☐ | ☒ Eukaryotic cell lines |
| ☒ | ☐ Palaeontology |
| ☒ | ☐ Animals and other organisms |
| ☒ | ☐ Human research participants |

## Methods

| n/a | Involved in the study |
|---|---|
| ☒ | ☐ ChIP-seq |
| ☒ | ☐ Flow cytometry |
| ☒ | ☐ MRI-based neuroimaging |

## Antibodies

| | |
|---|---|
| Antibodies used | anti-Flag M2 antibody (Sigma, F1804); anti-α tubulin antibody (Abcam, ab4074); goat anti-mouse HRP-conjugated antibody (Dako, P0447) |
| Validation | A very robust monoclonal Flag-antibody was used that had not to be further validated. The specificity of this antibody is very high, no cross reactivity was observed. The same is true for the polyclonal alpha Tubulin antibody (loading control). |

## Eukaryotic cell lines

Policy information about cell lines

| | |
|---|---|
| Cell line source(s) | HEK293T cells were originally purchased from ATCC (CRL-3216). |
| Authentication | HEK293T cells were only visually authenticated. |
| Mycoplasma contamination | The HEK293T cell line was not tested for mycoplasma contamination. |
| Commonly misidentified lines (See ICLAC register) | HEK293T cells served as a tool to investigate translation of transfected, exogenous mRNAs. To the best of our knowledge, there is no commonly misidentified cell line. |
